# Multi-omics analysis uncovers clinical, immunological, and pharmacogenomic implications of cuproptosis in clear cell renal cell carcinoma

**DOI:** 10.1186/s40001-023-01221-4

**Published:** 2023-07-22

**Authors:** Maoshu Zhu, Yongsheng Li, Yun Wang, Pingli Lin, Jun Mi, Weimin Zhong

**Affiliations:** The Fifth Hospital of Xiamen, Xiamen, 361101 Fujian People’s Republic of China

**Keywords:** Cuproptosis, Clear cell renal cell carcinoma, Prognosis, Immunogenomics, Immunotherapy, Precision therapy

## Abstract

**Objective:**

The latest research proposed a novel copper-dependent programmed cell death named cuproptosis. We aimed to elucidate the influence of cuproptosis in clear cell renal cell carcinoma (ccRCC) from a multi-omic perspective.

**Methods:**

This study systematically assessed mRNA expression, methylation, and genetic alterations of cuproptosis genes in TCGA ccRCC samples. Through unsupervised clustering analysis, the samples were classified as different cuproptosis subtypes, which were verified through NTP method in the E-MTAB-1980 dataset. Next, the cuproptosis score (Cuscore) was computed based on cuproptosis-related genes via PCA. We also evaluated clinical and immunogenomic features, drug sensitivity, immunotherapeutic response, and post-transcriptional regulation.

**Results:**

Cuproptosis genes presented multi-layer alterations in ccRCC, and were linked with patients’ survival and immune microenvironment. We defined three cuproptosis subtypes [C1 (moderate cuproptosis), C2 (low cuproptosis), and C3 (high cuproptosis)], and the robustness and reproducibility of this classification was further proven. Overall survival was best in C3, moderate in C1, and worst in C2. C1 had the highest sensitivity to pazopanib, and sorafenib, while C2 was most sensitive to sunitinib. Furthermore, C1 patients benefited more from anti-PD-1 immunotherapy. Patients with high Cuscore presented the notable survival advantage. Cuscore was highly linked with immunogenomic features, and post-transcriptional events that contributed to ccRCC development. Finally, several potential compounds and druggable targets (NMU, RARRES1) were selected for low Cuscore group.

**Conclusion:**

Overall, our study revealed the non-negligible role of cuproptosis in ccRCC development. Evaluation of the cuproptosis subtypes improves our cognition of immunogenomic features and better guides personalized prognostication and precision therapy.

**Supplementary Information:**

The online version contains supplementary material available at 10.1186/s40001-023-01221-4.

## Introduction

Renal cell carcinoma (RCC) originating from renal tubular epithelial cells occupies ~ 3.8% of all newly diagnosed cancers [1]. Clear cell RCC (ccRCC) is an aggressive histological subtype that accounts for ~ 75% of all cases [2]. Over one-third of ccRCC patients experience relapse and metastasis after surgery, and patients with metastatic ccRCC have an undesirable prognosis, with a 5-year survival rate of 10% [3]. Currently, the effects of ccRCC clinical treatment modalities mainly incorporating surgical management, conventional chemotherapy, targeted therapy, and immunotherapy, etc., is limited by intratumoral heterogeneity that challenges the molecular characterization of ccRCC and is a confounding factor for treatment selection [4, 5]. Recently, multi-omics technology have led to a significant advantage in understanding the molecular mechanism and cancer management [6, 7]. Extensive genomic characterization has unveiled a few genetic alterations (VHL, PBRM1, etc.) correlated to ccRCC [8]. Moreover, large-scale molecular profiling analysis (The Cancer Genome Atlas (TCGA), etc.) has identified critical biological processes in ccRCC [9]. Nonetheless, molecular profiling is not routinely applied for ccRCC prognostication and therapeutic options. Additional molecular mechanisms may further improve stratification of ccRCC patients into proper risk classifications.

**Fig. 1:**
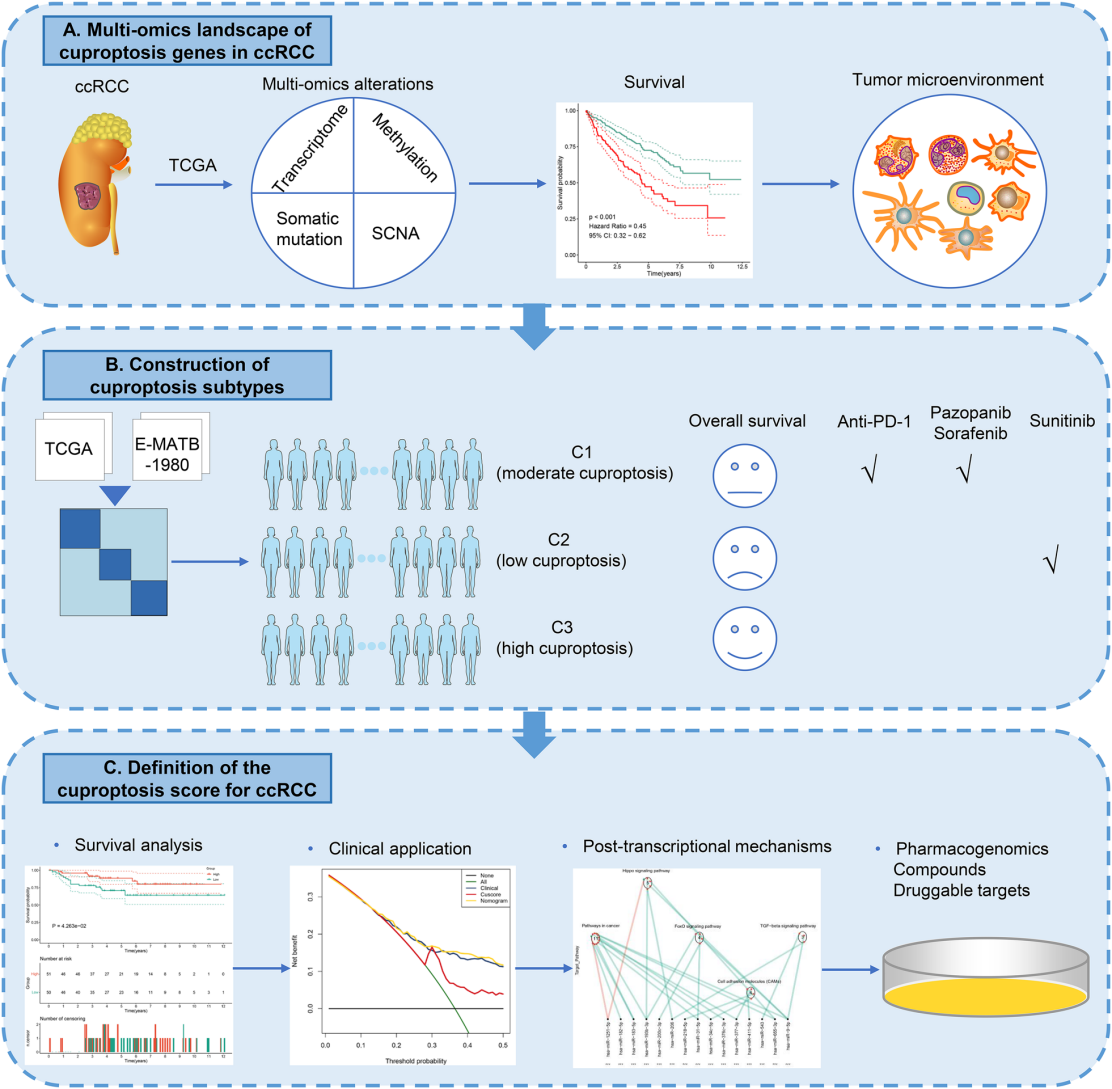
Schematic diagram of the study design

Copper is a fundamental mineral nutrient, and its redox properties make it both beneficial and toxic to cells [10]. Both serum and tumor copper levels are elevated in solid tumors, which directly correlate to cancer progression [11]. Recently, a novel copper-dependent programmed cell death mechanism named cuproptosis has been proposed, which strongly correlates to mitochondrial metabolism and is mediated by protein lipoylation [12]. Evidence demonstrates that cuproptosis genes exhibit aberrant expression in ccRCC and are notably linked with patients’ prognosis [13]. In addition, cuproptosis might potentially predict immunotherapeutic response of ccRCC [14]. However, our cognition of cuproptosis is still in its infancy. Molecular profiling enables to provide crucial prognostic information and treatment guidance for the management of ccRCC patients. In this study, the clinical, immunological and pharmacogenomic role of cuproptosis was investigated in ccRCC, which may shed light on optimizing the precision management of ccRCC patients. The flowchart of our research is illustrated in Fig. 1.

## Materials and methods

### Publicly available ccRCC datasets

Transcriptome profiling (HTSeq-counts) of ccRCC (*n* = 539) and normal (*n* = 72) specimens together with clinical parameters were acquired from TCGA (https://portal.gdc.cancer.gov/). Raw counts data were converted into transcripts per million (TPM), followed by log2 transformation. DNA methylation data (Methylation450K), somatic mutation (mutation annotation format), copy-number alteration (SCNA; Masked Copy Number Segment) and microRNA (miRNA) expression data of ccRCC patients were also collected from TCGA. Matrix files of transcriptome profiling and prognostic data of ccRCC patients (*n* = 101) were acquired from the E-MTAB-1980 dataset (https://www.ebi.ac.uk/arrayexpress/experiments/E-MTAB-1980/), which was utilized for external verification. The detailed clinicopathological information is listed in Additional file 2: Table S1.

### Collection of cuproptosis genes

Cuproptosis genes were collected from a previously published study [15]. Circos plot containing chromosome positions of cuproptosis genes was drawn via RCircos package [12].

### Methylation analysis

For DNA methylation data, only the probes that mapped to the promoter region of cuproptosis genes were retained. For genes with multiple probes, the average β value of all probes was utilized as the methylation level.

### Somatic mutation and SCNA analysis

To lower the false-positive rate, only non-silent mutations were retained. Through maftools package [16], the mutation annotation format from TCGA was analyzed. For SCNA, significant amplifications and deletions were identified via GISTIC2.0 [17]. Then, OncoPrint plots of mutations and SCNA were produced with ComplexHeatmap package [18].

### Functional enrichment analysis

The enrichment score of Gene Ontology (GO) and Kyoto Encyclopedia of Genes and Genomes (KEGG) gene sets was evaluated between low and high expression of CDKN2A groups using gene set enrichment analysis (GSEA) [19]. GO and KEGG enrichment analysis of cuproptosis-related genes was conducted via clusterProfiler package [20]. Terms with false discovery rate (FDR) < 0.05 were regarded as significant enrichment. Gene set variation analysis (GSVA) [21], and fast GSEA (fGSEA) were used to compute and compare the activity of the 50 hallmark pathways based on the Molecular Signatures Database-derived “h.all.v7.4.entrez.gmt” gene set as the reference [22].

### Unsupervised clustering analysis

ccRCC samples were classified as distinct cuproptosis subtypes based on the transcriptome profiling of cuproptosis genes using unsupervised clustering algorithm. The number of clusters was identified via ConsensusClusterPlus package [23], with iterations for increasing the classification reliability.

### Subtype validation

The top 200 up-regulated genes in each subtype versus others were identified via limma package [24], which were used to validate the cuproptosis subtypes through nearest template prediction (NTP) algorithm in the E-MTAB-1980 cohort [25].

### Immunogenomic and stemness signatures

Single-nucleotide variant (SNV) neoantigens, tumor mutation burden (TMB), SCNA, cancer-testis antigen (CTA) score, homologous recombination defects, intratumor heterogeneity, and aneuploidy score were acquired from previously published literature [26]. In addition, mRNA expression-based stemness index (mRNAsi) was computed utilizing one-class logistic regression machine-learning approach [27].

### Drug sensitivity assessment

Drug sensitivity data of cancer cell lines (CCLs) were acquired from Genomics of Drug Sensitivity in Cancer (GDSC) [28], Cancer Therapeutics Response Portal (CTRP) [46], and PRISM [29] databases. GDSC covers the half-maximal inhibitory concentration (IC50) data; meanwhile, both CTRP and PRISM cover the area under the curve (AUC) data as an evaluation indicator of drug sensitivity. Transcriptome profiling of CCLs was acquired from the Cancer Cell Line Encyclopedia (CCLE) [30]. The IC50 of GDSC-derived compounds was evaluated through oncoPredict package [31]. CTRP- and PRISM-derived compounds with more than 20% missing data were removed before K-nearest neighbor imputation. Next, pRRophetic package was employed to estimate the AUC of compounds [32]. Spearman correlation analysis on Cuscore and IC50 or AUC was executed to evaluate the drug response of ccRCC patients to each compound.

### Tumor-infiltrating immune cells

Single-sample GSEA (ssGSEA) was utilized to quantify the relative abundance of 22 tumor-infiltrating immune cell types and two stromal components (fibroblasts and endothelial cells) based on the known marker genes (Additional file 3: Table S2) [33]. Immune score and stromal score were estimated through ESTIMATE algorithm [34].

### Multi‑omics analysis of immunomodulators

Multi-omics profiling (comprising mRNA expression, SCNA, and DNA methylation) of 75 immunomodulators was observed across distinct cuproptosis subtypes (Additional file 4: Table S3) [35].

### Immunotherapy response prediction

Subclass Mapping (SubMap) analysis was adopted to assess the expression similarity between the three cuproptosis subtypes and immunotherapeutic responses [36]. The degree of commonality between the two groups was inferred via GSEA algorithm. Bonferroni-corrected *p* < 0.05 denoted that two groups were significantly similar.

### Generation of the cuproptosis score (Cuscore)

To identify the cuproptosis-related genes based on the three cuproptosis subtypes, differentially expressed genes (DEGs) were screened through limma package with the threshold of |fold change|> 1.5 together with adjusted *p* < 0.05. Prognostic value of cuproptosis-related genes was assessed via univariate Cox regression analysis. Then, genes with *p* < 0.05 were retained for computing the Cuscore through adopting PCA, with the principal components (PCs) 1 and 2 as the final signature score. The Cuscore was defined as Cuscore = $\sum_{n}^{m}(PCn+PCm)$, where n and m denoted the order and total number of prognostic cuproptosis-related genes in ccRCC, respectively.

### Nomogram construction

Univariate Cox regression on clinicopathological parameters together with Cuscore was analyzed across ccRCC patients. Thereafter, significant prognostic predictors (*p* < 0.05) were retained for multivariate Cox regression analysis, and a nomogram was defined through adopting independent predictive variables (*p* < 0.05). The consistency between predicted and actual prognostic outcome was assessed via calibration curves. Through decision curve analysis, the net benefits of the nomogram, Cuscore and other clinicopathological parameters were measured.

### Analysis of post-transcriptional mechanisms

MiRNAs with differential expression were screened between low and high Cuscore groups based on |fold change|> 2 and adjusted *p* < 0.01. Then, targeted pathways were enriched through KEGG enrichment analysis.

### Statistical analysis and visualization

Continuous variables in two or more than two groups were compared utilizing parametric test (Student’s t-test or analysis of variance) if the variables displayed normal distribution or nonparametric test (Wilcoxon rank-sum test or Kruskal–Wallis test). Associations between continuous variables were evaluated via Pearson or Spearman correlation test. Kaplan–Meier (K-M) analysis of overall survival (OS) with log-rank test was conducted using survminer package. Time-dependent receiver operating characteristic (ROC) curves were plotted with timeROC package, and AUC was computed. Relationships of Cuscore with clinicopathological parameters were analyzed through Chi-square test. The discrimination of transcriptome profiling between groups was displayed via principal component analysis (PCA). Uni- and multivariate Cox regression analysis was executed to determine the prognostic genes and independent prognostic parameters via survival package. All statistical analyses were executed through R packages. Statistical significance was set as a two-tailed *p* < 0.05.

## Results

### Multi-omics landscape of cuproptosis genes in ccRCC

**Fig. 2:**
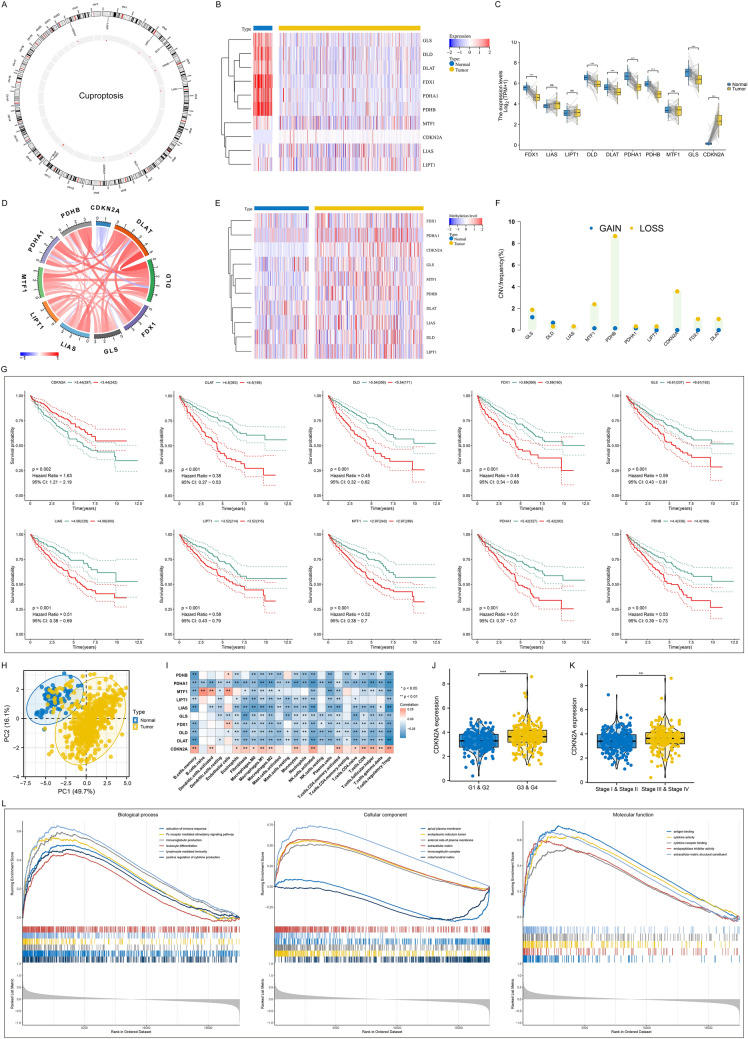
Multi-omics landscape of cuproptosis genes across ccRCC in TCGA cohort. **A** Circos plot shows the chromosome positions of cuproptosis genes. **B** Transcriptome profiling of cuproptosis genes in ccRCC versus normal specimens. Colors from blue to red denote low to high expression. **C** Comparison of cuproptosis genes in paired ccRCC and normal specimens. The center line indicates the median, and the upper and lower lines indicate the upper and lower quartiles. **D** Interactions between cuproptosis genes at the transcriptional level. Blue line, negative correlation; red line, positive correlation. **E** Methylation profiling of cuproptosis genes in ccRCC versus normal specimens. Colors from blue to red indicate low to high methylation. **F** Frequencies of copy number amplification and deletion of cuproptosis genes in ccRCC. Blue dot, amplification; yellow dot, deletion. **G** K-M curves for OS between groups separated by the median expression value of each cuproptosis gene. **H** PCA plots illustrate the discrimination of transcriptome profiling of cuproptosis genes between ccRCC and normal samples. Each dot denotes a sample. Blue dot, normal tissue; yellow dot, ccRCC tissue. **I** Associations of cuproptosis genes with 22 tumor-infiltrating immune cell types and two stromal cells within the tumor microenvironment. Colors from blue to red denote negative to positive correlation. **J**, **K** Comparison of the transcription levels of CDKN2A between groups separated by grade and stage. Each point indicates a sample; the center line indicates the median, and the upper and lower lines indicate the upper and lower quartiles. **L** GSEA shows the GO terms with significance differences between low and high CDKN2A expression groups. For asterisks, ns: p-value > 0.05; *p < 0.05; **p < 0.01; ***p < 0.001

Figure 2A illustrates the genomic location of each cuproptosis gene. Most cuproptosis genes (FDX1, DLD, DLAT, PDHA1, PDHB, and GLS) were notably down-regulated in ccRCC versus normal tissues, with only up-regulated CDKN2A (Fig. 2B, C). At the transcriptional level, cuproptosis genes positively interacted except CDKN2A in ccRCC (Fig. 2D). Next, we analyzed the reasons for the low expression of cuproptosis genes. Higher methylation levels of most cuproptosis genes were observed in ccRCC than normal tissues (Fig. 2E). In addition, copy number deletion of cuproptosis genes occurred in ccRCC, especially PDHB (Fig. 2F). Hypermethylation and copy number deletion might contribute to the low expression of cuproptosis genes. Their prognostic value was then assessed. Consequently, most cuproptosis genes were linked with better OS outcome of ccRCC, with opposite effect of CDKN2A (Fig. 2G). PCA revealed the remarkable discrimination of transcriptome profiling of cuproptosis genes between ccRCC and normal samples (Fig. 2H). Altogether, cuproptosis might be indispensable for ccRCC initiation and progression. ccRCC is highly immune infiltrated; adaptive and innate immune cells infiltrate the tumor microenvironment and constitute an ecosystem that regulates each aspect of ccRCC development [37]. Most cuproptosis genes were negatively correlated to the infiltration of immune cells, with positive relationships between CDKN2A and immune cell infiltration (Fig. 2I). Based on above findings, CDKN2A was identified as a cuproptosis gene of interest. CDKN2A expression was higher in more advanced grade and stage (Fig. 2J, K). GSEA revealed that CDKN2A positively correlated to immunity (activation of immune response, immunoglobulin production, lymphocyte-mediated immunity, etc.) (Fig. 2L).

### Construction and external verification of cuproptosis subtypes in ccRCC

**Fig. 3:**
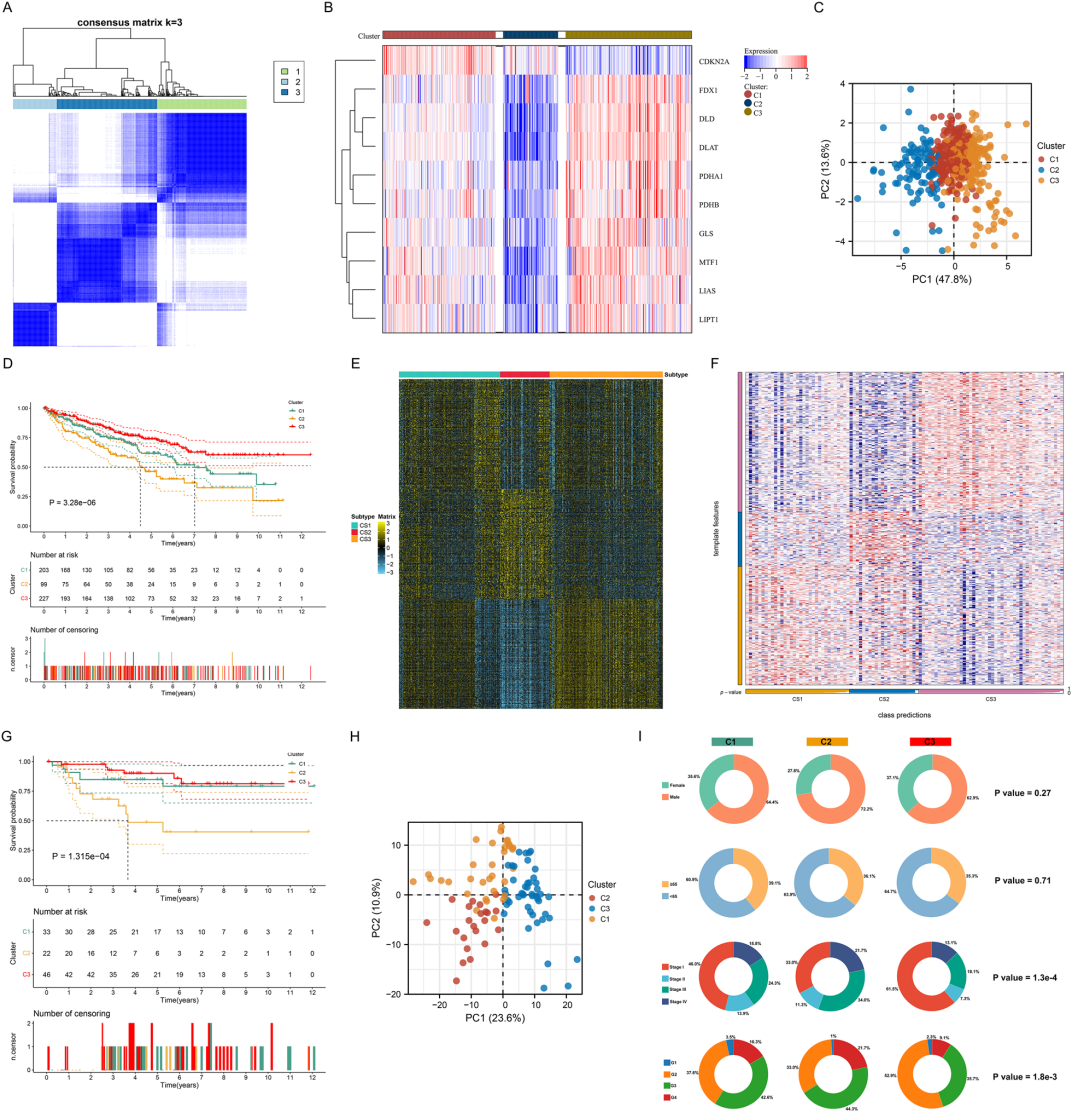
Construction and external verification of cuproptosis subtypes in ccRCC. **A** Consensus matrix depicts the consensus value *k* = 3 on a white to blue color scale based on the transcriptome profiling of cuproptosis genes in TCGA cohort. **B** Transcriptome profiling of cuproptosis genes in the three cuproptosis subtypes in TCGA cohort. Colors from blue to red display low to high expression. **C** PCA exhibits the discrimination of transcriptome profiling of cuproptosis genes among the three cuproptosis subtypes in TCGA cohort. **D** K-M curves for OS of the three cuproptosis subtypes in TCGA cohort. **E** Transcriptome profiling of up-regulated genes in each cuproptosis subtype in accordance with fold change > 1 and adjusted *p* < 0.05. **F** Transcriptome profiling of the template features in the cuproptosis subtypes in the E-MATB-1980 dataset. **G** Validation of OS difference among the three cuproptosis subtypes in the E-MATB-1980 cohort. **H** Validation of the discrimination of transcriptome profiling among the three cuproptosis subtypes via PCA in the E-MATB-1980 cohort. **I** Proportions of clinicopathological parameters in each cuproptosis subtype across TCGA ccRCC patients

According to the transcriptome profiling of cuproptosis genes, cuproptosis subtypes were established via unsupervised clustering analysis. When the consensus value *k* was 3, most of the colors in the consensus matrix did not overlap (Fig. 3A). Cumulative distribution function (CDF) as well as item tracking demonstrated the cluster stability when *k* = 3 (Additional file 1: Figure S1A–C). Taken together, we classified ccRCC as three cuproptosis subtypes, namely C1 (moderate cuproptosis), C2 (low cuproptosis), and C3 (high cuproptosis) (Additional file 5: Table S4; Fig. 3B). PCA illustrates the notable discrepancy of transcriptome profiling among the three cuproptosis subtypes (Fig. 3C). Prognosis difference was observed among the three cuproptosis subtypes, with the best OS in C3, intermediate in C1, and worst in C2 (Fig. 3D). According to fold change > 1 together with adjusted *p* < 0.05, we determined up-regulated genes in each cuproptosis subtype (Fig. 3E). The top 200 genes were regarded as up-regulated biomarkers of each cuproptosis subtype (Additional file 6: Table S5). To further verify the stability and robustness of cuproptosis subtypes, NTP analysis that may quantify the prediction confidence of each patient based on transcriptome profiling was implemented in the E-MATB-1980 cohort. Consequently, the three cuproptosis subtypes exhibited high reproducibility in the E-MATB-1980 dataset (Fig. 3F). The differences in OS outcome and transcriptome profiling among the three cuproptosis subtypes were proven in this cohort (Fig. 3G, H). Clinicopathological features were prominently different among the three cuproptosis subtypes, with the lowest proportions of grade and stage in C3, intermediate in C1, and highest in C2 (Fig. 3I). Altogether, the cuproptosis-based classification was reproducible and stable in ccRCC.

### Genomic alterations, stemness, and drug sensitivity features across cuproptosis subtypes

**Fig. 4:**
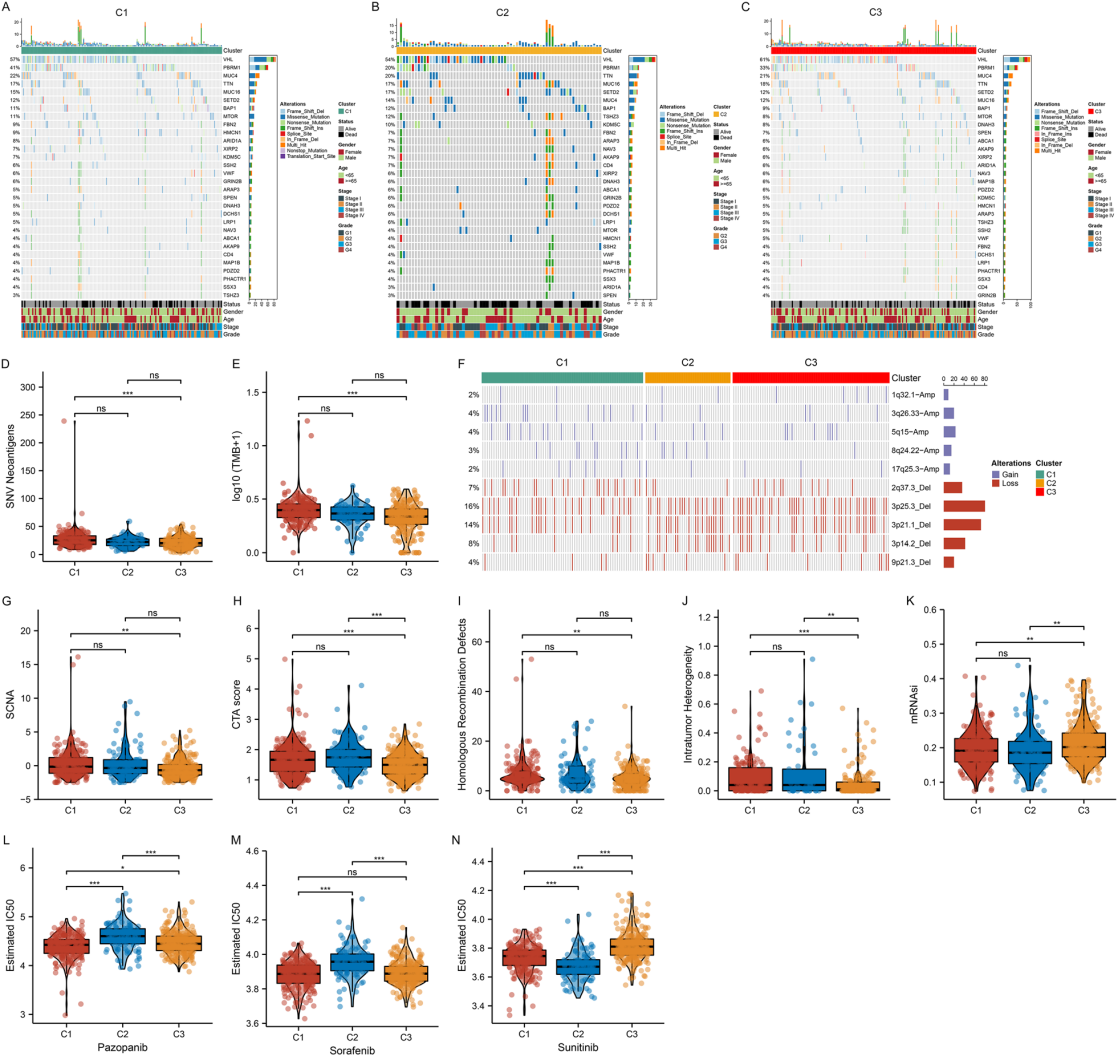
Genomic alterations, stemness, and drug sensitivity features across cuproptosis subtypes in TCGA cohort. **A**–**C** OncoPrint plots show the top 30 genes with mutation frequencies across cuproptosis subtypes. Row denotes mutated genes, and column denotes ccRCC samples. The left bar exhibits the mutation percentage, and the top bar exhibits the total number of mutations. Clinicopathological features are displayed at the bottom. **D**, **E** Differences in SNV neoantigens and TMB between cuproptosis subtypes. **F** OncoPrint plot exhibits the top 5 copy number amplifications and deletions across cuproptosis subtypes. Row denotes SCNA, and column denotes ccRCC samples. The left bar displays the percentage of SCNA, and the right bar displays the total number of SCNA. **G**–**K** Differences in SCNA, CTA score, homologous recombination defects, intratumor heterogeneity, and mRNAsi between cuproptosis subtypes. **L**–**N** Differences in IC50 of GDSC-derived compounds between cuproptosis subtypes. For violin plots, each point denotes a sample; the center line denotes the median, and the upper and lower lines denote the upper and lower quartiles. For asterisks, ns: *p* > 0.05; *p < 0.05; **p < 0.01; ***p < 0.001

DNA alterations mainly comprise somatic mutation and SCNA. Overall, somatic mutation exhibited the remarkable heterogeneity among the three cuproptosis subtypes (Fig. 4A–C). Of note, C3 had the highest mutation frequency of VHL (61%), followed by C1 (57%) and C2 (54%). PBRM1 had the highest mutation frequency in C1 (41%), intermediate in C3 (33%), and lowest in C2 (20%). Overall, C3 presented significantly lower SNV neoantigens and TMB score relative to C1 (Fig. 4D, E). Next, we observed the heterogeneity of SCNA profiling in the three cuproptosis subtypes (Fig. 4F). Additionally, C1 presented higher SCNA, CTA score, homologous recombination defects, and intratumor heterogeneity (Fig. 4G–J). We computed mRNAsi for quantifying cancer stemness, and found that C3 had higher mRNAsi (Fig. 4K). Drug sensitivity differences were also assessed among the three cuproptosis subtypes. Consequently, C1 patients had the highest sensitivity to pazopanib, and sorafenib, while C2 patients were most sensitive to sunitinib (Fig. 4L–N).

### Three cuproptosis subtypes with distinct immunotherapeutic responses

**Fig. 5:**
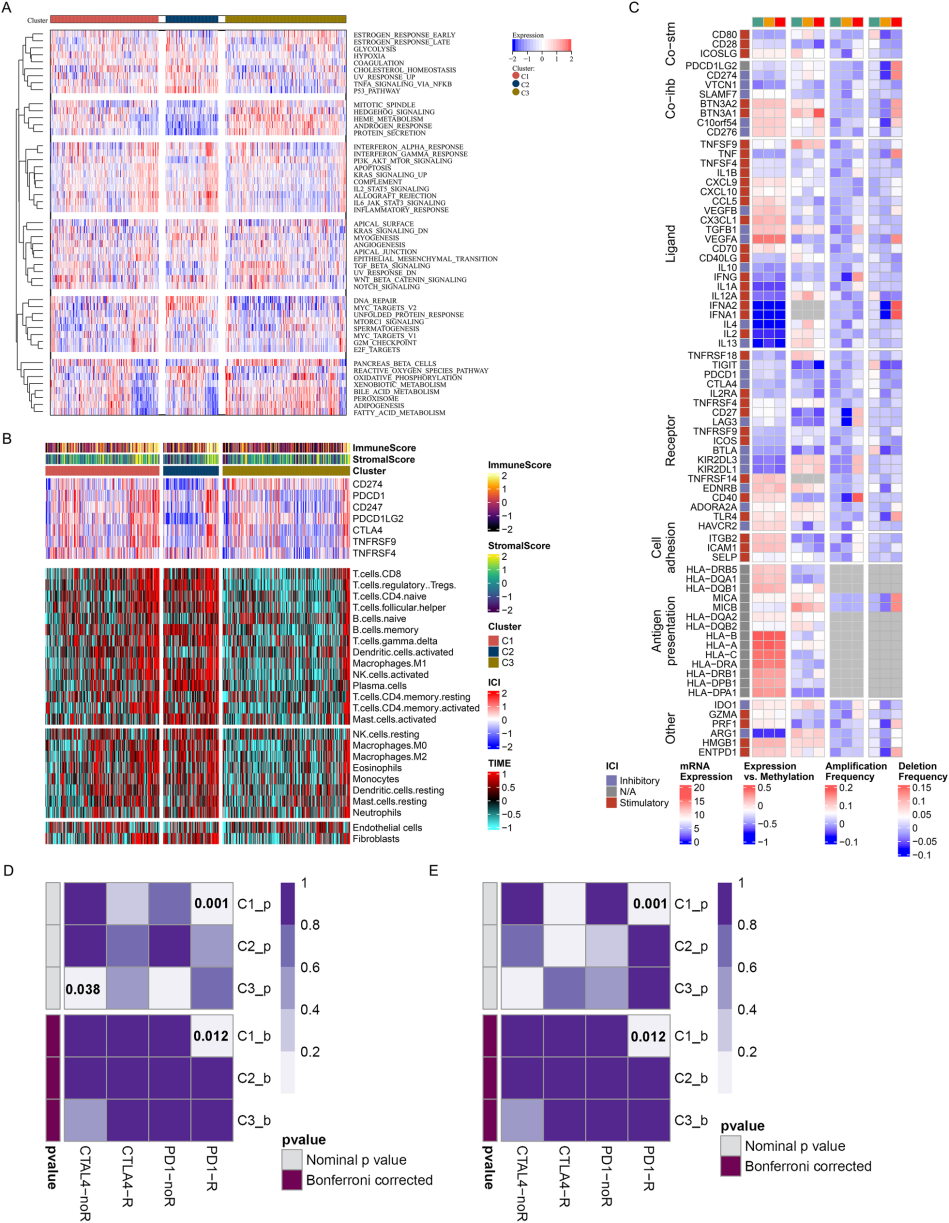
Three cuproptosis subtypes with distinct immunotherapeutic responses. **A** The relative activity status of the 50 hallmark pathways in the three cuproptosis subtypes in TCGA cohort. Colors from blue to red denote low to high activity. **B** Landscape of immune score, and stromal score, mRNA expression of common immune checkpoints, relative abundance of 22 tumor-infiltrating immune cell types and two stromal components in TCGA cohort. **C** Multi-omics profiling (mRNA expression, DNA methylation level, and amplification/deletion frequency) of 75 immunomodulators in TCGA cohort. **D** SubMap analysis shows the expression similarity between the three cuproptosis subtypes and responses to anti-PD-1 and anti-CTLA4 in TCGA cohort. **E** Validation of the expression similarity between the three cuproptosis subtypes and responses to anti-PD-1 and anti-CTLA4 in the E-MTAB-1980 dataset

Next, we observed the remarkable differences in the oncogenic hallmark pathways among the three cuproptosis subtypes, contributing to the intratumor heterogeneity of ccRCC (Fig. 5A). C1 subtype presented the highest expression of common immune checkpoint molecules (PDCD1, CTLA4, etc.) (Fig. 5B). Additionally, the most abundant infiltration of immune cells was observed in C2, with intermediate for C1, and lowest for C3. In Fig. 5C, there were extensive genomic differences in immunomodulators across the three cuproptosis subtypes. Of note, C3 had the highest frequencies of amplification (IFNG, CD27, LAG3, CD40, GZMA, etc.) and deletion (PDCD1LG2, CD274, BTN3A1, BTN3A2, TNF, VEGFA, IFNA1, IFNA2, MICA, MICB, TLR4, PRF1, ENTPD1, etc.) of several immunomodulators, followed by C1 and C2. Considering that the cuproptosis subtypes appear to correlate to the tumor immune microenvironment, we inferred the immunotherapeutic responses of the three cuproptosis subtypes. Consequently, C1 subtype exhibited the high expression similarity to response to anti-PD-1 therapy (Fig. 5D), proven in the E-MTAB-1980 dataset (Fig. 5E). Hence, patients in C1 subtype clinically benefited more from anti-PD-1 immunotherapy.

### Definition of the Cuscore for individual ccRCC patients

**Fig. 6:**
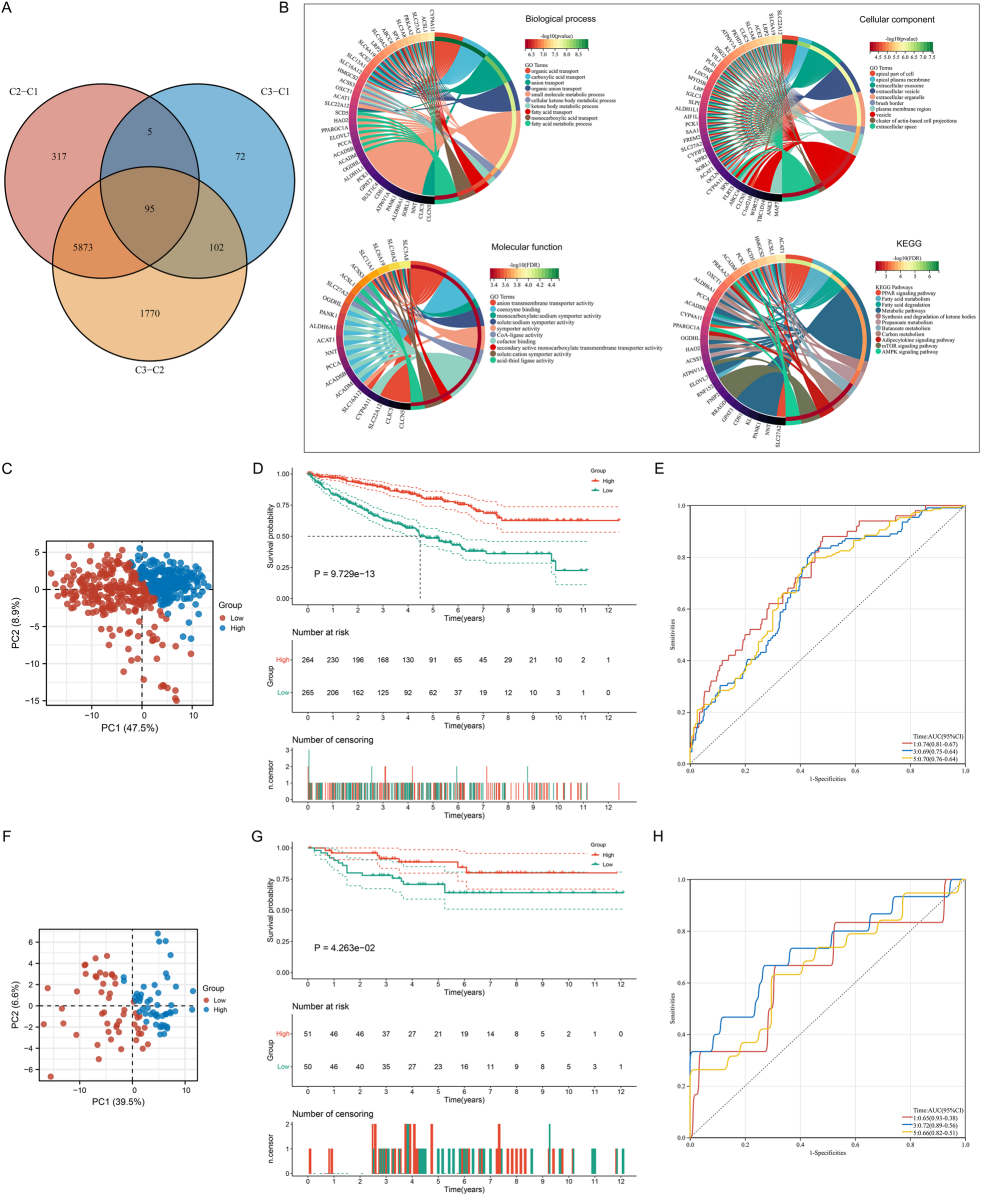
Definition of the Cuscore reliably predicting ccRCC prognosis. **A** Venn diagram shows the intersection of DEGs between cuproptosis subtypes in TCGA cohort. **B** GO and KEGG analysis results of cuproptosis-related genes. **C** PCA illustrates the discrimination of transcriptome profiling between low and high Cuscore groups in TCGA cohort. Each dot denotes a sample. **D** K-M curves of OS between groups in TCGA cohort. **E** ROC curves of 1-, 3- and 5-year OS based on the Cuscore in TCGA cohort. **F** Validation of the distinction of transcriptome profiling between low and high Cuscore groups in the E-MTAB-1980 cohort. **G** K-M curves of OS between groups in the E-MTAB-1980 dataset. **H** ROC curves of 1-, 3- and 5-year OS for the Cuscore in the E-MTAB-1980 cohort

To assess the functional role of the three cuproptosis subtypes, 95 cuproptosis-related genes were determined (Fig. 6A; Additional file 7: Table S6) through intersecting DEGs between cuproptosis subtypes according to |fold change|> 1.5 and adjusted p < 0.05. Functional enrichment analysis revealed that they were remarkably enriched in diverse metabolism processes (Fig. 6B). Next, the prognostic value of cuproptosis-related genes was investigated. Consequently, all of them were significantly linked with ccRCC patients’ OS (Additional file 8: Table S7). Through PCA computational approach, the Cuscore was defined based on cuproptosis-related genes to quantify cuproptosis of individual ccRCC patients. Next, we investigated the clinical relevance of the Cuscore. ccRCC patients were classified as low and high Cuscore groups, with the prominent distinction of transcriptome profiling (Fig. 6C). Patients with high Cuscore possessed a remarkable survival benefit (Fig. 6D), with 1-, 3- and 5-year OS AUC values of 0.74, 0.69 and 0.70, respectively (Fig. 6E). The E-MTAB-1980 dataset proved the high reproducibility of this Cuscore (Fig. 6F–H).

### Associations of the Cuscore with clinicopathological characteristics

**Fig. 7:**
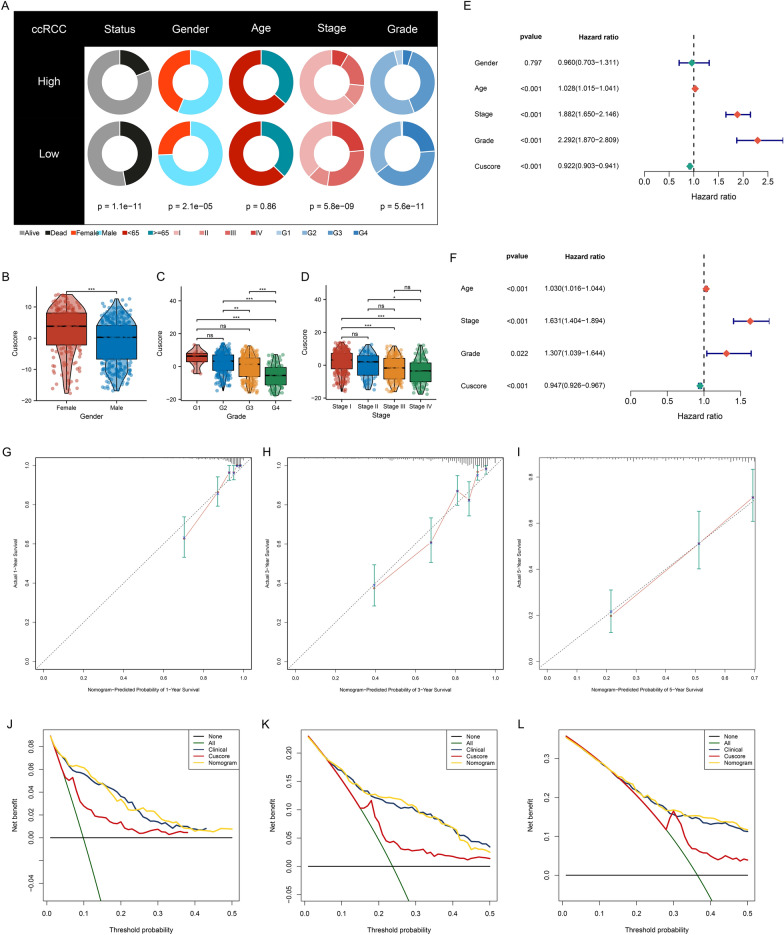
Associations of the Cuscore with clinicopathological features and definition of the Cuscore-based nomogram for prognostic prediction in TCGA cohort. **A** Clinicopathological features of low and high Cuscore groups. **B**–**D** Cuscore differences in distinct gender, grade, and stage. For asterisks, ns: *p* > 0.05; *p < 0.05; **p < 0.01; ***p < 0.001. **E**, **F** Uni- and multivariate Cox regression analysis of clinicopathological parameters and Cuscore on OS outcome. **G**–**I** Calibration curves show the consistency between the nomogram-predicted and actual prognostic outcome. **J**–**L** Decision curve analysis evaluates the net benefits of ccRCC patients from the nomogram, Cuscore and other clinicopathological parameters

Next, clinicopathological characteristics of low and high Cuscore groups were observed. In Fig. 7A, low Cuscore patients exhibited higher proportions of dead status, male, advanced stage, and grade. Additionally, male patients had lower Cuscore relative to female patients (Fig. 7B). With the increase in grade and stage, the Cuscore gradually lowered (Fig. 7C, D). Altogether, the Cuscore may reflect tumor progression and prognostic outcome.

### Generation of the Cuscore-based nomogram for predicting ccRCC prognosis

From uni- and multivariate Cox regression results, Cuscore served as an independent protective factor, with clinicopathological variables (age, stage, and grade) as independent risk factors (Fig. 7E, F). To facilitate the clinical utility of the Cuscore in predicting the survival probability, we generated a nomogram that integrated above independent prognostic factors. Calibration curves of 1-, 3- and 5-year OS displayed that the nomogram was close to the actual survival probability (Fig. 7G–I). Moreover, we found that the nomogram had the highest net benefit in clinical assessment based on decision curve analysis, suggesting the significance of the Cuscore in coordination with other clinicopathological variables for survival prediction (Fig. 7J–L).

### Immunogenomic features, and oncogenic hallmark pathways associated with Cuscore

**Fig. 8:**
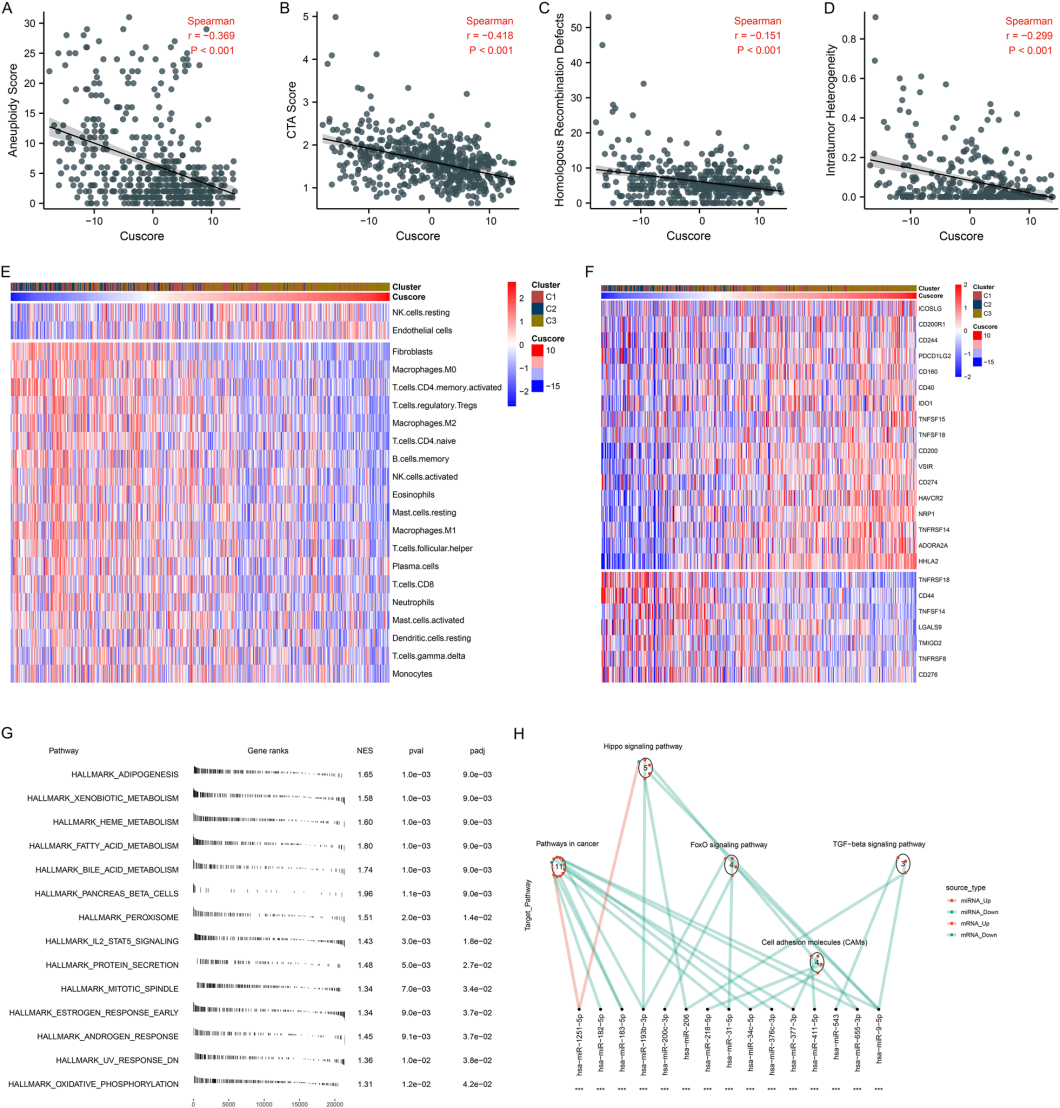
Associations of Cuscore with immunogenomic features, oncogenic hallmark pathways, post-transcriptional mechanisms in TCGA cohort. **A**–**D** Spearman correlation analysis of Cuscore with aneuploidy score, CTA score, homologous recombination defects, and intratumor heterogeneity. **E** Relative abundance of 22 tumor-infiltrating immune cell types and two stromal components in ccRCC samples with low to high Cuscore. Colors from blue to red correspond to low to high cell abundance. **F** Transcriptome profiling of immune checkpoints across ccRCC samples with low to high Cuscore. Colors from blue to red correspond to low to high expression of immune checkpoints. **G** The fGSEA compares the activity of the hallmark pathways between low and high Cuscore groups. **H** Differences in miRNA-targeted pathways between low and high Cuscore samples. Red line denotes a down-regulated miRNA in high Cuscore group, and cyan line denotes an up-regulated miRNA. Red dot represents a miRNA-targeted mRNA up-regulated in high Cuscore group, and cyan dot represents a down-regulated miRNA-targeted mRNA. Circle corresponds to a pathway enriched by targeted mRNAs. For asterisks, ***p < 0.001

To understand the immunogenomic features involved in the Cuscore, we evaluated the relationships of the Cuscore with immunogenomic signatures. Consequently, the Cuscore was negatively linked with aneuploidy score, CTA score, homologous recombination defects, and intratumor heterogeneity in ccRCC (Fig. 8A–D). Additionally, we observed the negative associations of the Cuscore with most immune cells (Fig. 8E), and most immune checkpoint molecules were up-regulated as the Cuscore increased (Fig. 8F). The fGSEA revealed that the Cuscore was prominently correlated to oncogenic hallmark pathways (Fig. 8G).

### Post-transcriptional mechanisms involved in Cuscore

Next, this study analyzed miRNAs with differential expression between low and high Cuscore groups. Sixty-nine miRNAs that presented significant differential expression were selected (Additional file 9: Table S8), and KEGG pathway enrichment analysis of their targeted mRNAs was implemented. Consequently, pathways in cancer, cell adhesion molecules (CAMs), Hippo, FoxO and TGF-β signaling pathways were notably enriched (Fig. 8H). Most miRNA-targeted mRNAs in above pathways were up-regulated in high Cuscore group. Above evidence suggested that Cuscore was linked with post-transcriptional mechanisms and pathway regulation.

### Identification of Cuscore-related compounds

**Fig. 9:**
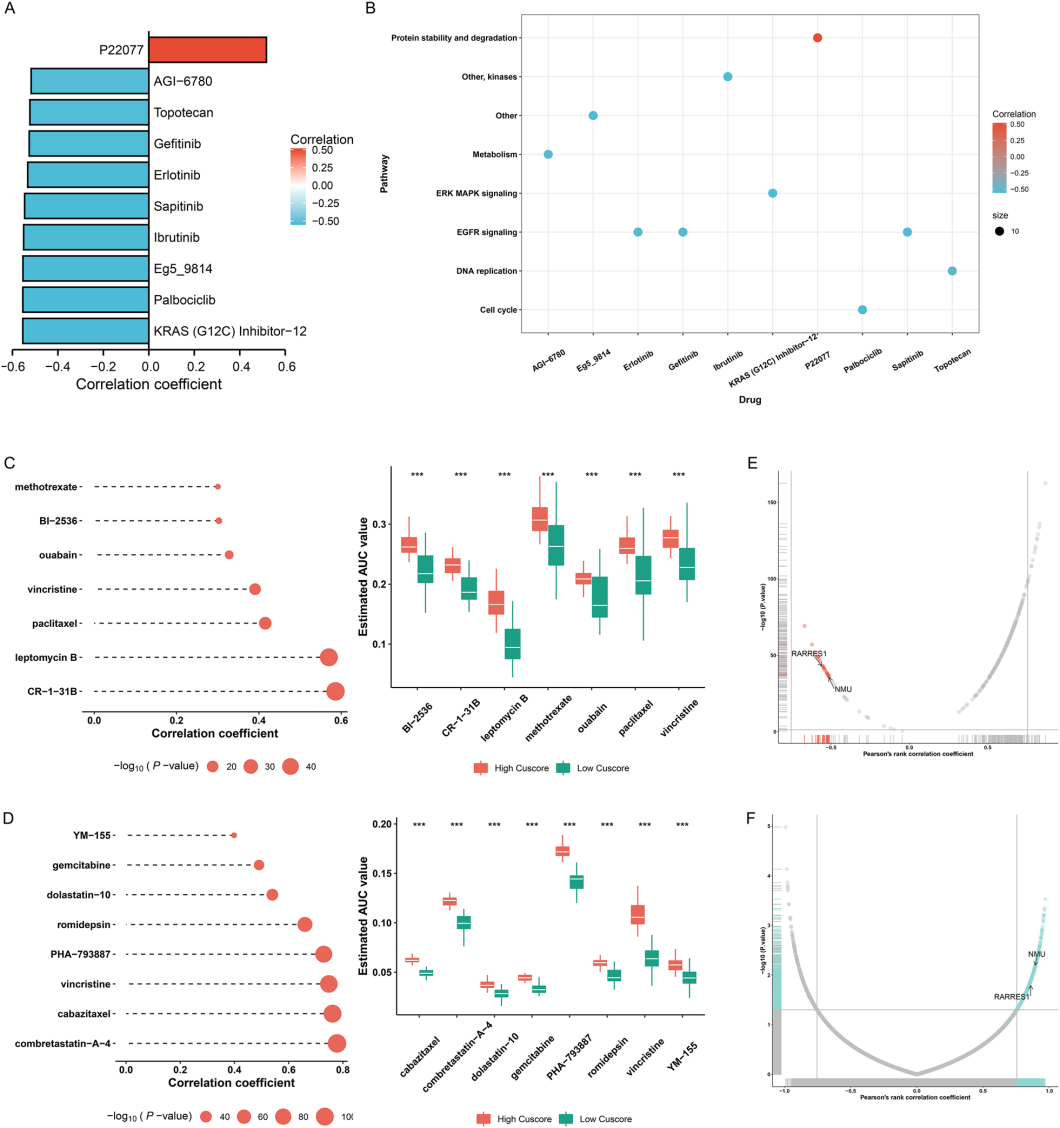
Recognition of Cuscore-relevant compounds and druggable targets. **A** Associations of Cuscore with estimated IC50 values of compounds from GDSC. **B** Pathways underlying GDSC-derived compounds. **C** Associations of Cuscore with estimated AUC values of compounds from CTRP (left), and comparison of AUC values of compounds between low and high Cuscore groups (right). **D** Spearman correlation analysis on Cuscore and estimated AUC values of PRISM-derived compounds (left), and comparison of AUC values of compounds between low and high Cuscore groups (right). **E** Relationships of Cuscore with protein expression of druggable targets. Red dot denotes a significant negative correlation (correlation coefficient < −0.5 and *p* < 0.05). **F** Relationships of Cuscore with CERES score of druggable targets. Blue dot denotes a significant negative correlation (correlation coefficient > 0.75 and *p* < 0.05)

We further understood the effects of the Cuscore on drug response. The associations of the Cuscore with the response to GDSC-derived compounds were then assessed. The sensitivity to nine drugs (AGI-6780, topotecan, gefitinib, erlotinib, sapitinib, ibrutinib, Eg5_9814, palbociclib and KRAS (G12C) Inhibitor-12) correlated to the high Cuscore, with the correlation between P22077 resistance and the high Cuscore (Fig. 9A). Next, we investigated the pathways involved in these drugs. As illustrated in Fig. 9B, drugs whose sensitivity was linked with the high Cuscore were mainly targeting cell cycle, DNA replication, EGFR and ERK MAPK signaling. In addition, the CTRP and PRISM databases were utilized to predict potential compounds for ccRCC. CR-1-31B, leptomycin B, paclitaxel, vincristine, ouabain, BI-2536, methotrexate, combretastatin-A-4, cabazitaxel, vincristine, PHA-793887, romidepsin, dolastatin-10, gemcitabine, and YM-155 were more suitable for patients with low Cuscore (Fig. 9C, D). Altogether, our findings implied that cuproptosis was correlated to drug response. Therefore, the Cuscore might be a potential biomarker for formulating appropriate therapeutic schedule.

### Identification of potential druggable targets for patients with low Cuscore

Potentially druggable targets for patients with low Cuscore were identified. Firstly, correlation between the Cuscore and protein expression of druggable targets was computed, and 23 protein targets were selected according to correlation coefficient < −0.5 and *p* < 0.05 (Fig. 9E). Then, we conducted correlation analysis of CERES score with the Cuscore, and further screened 486 targets based on correlation coefficient > 0.75 and *p* < 0.05 (Fig. 9F). Two genes (NMU, RARRES1) were finally determined as potential therapeutic targets by above methods.

## Discussion

Copper is a trace metal in cells that is indispensable for maintaining the function of proteins. Nonetheless, excess copper can result in cytotoxicity [38]. Zvetkov et al. firstly proposed a copper accumulation-dependent cuproptosis, which is distinct from other cell death mechanisms [12]. Renal cell carcinoma (RCC) is essentially a metabolic disease characterized by a reprogramming of energetic metabolism [39–42]. In particular the metabolic flux through glycolysis is partitioned [43–45], and mitochondrial bioenergetics and OxPhox are impaired, as well as lipid metabolism [43, 46–48]. Moreover, accumulative researches have been demonstrated that cuproptosis play an important role as regulators of cell metabolism [49–51]. Therefore, this study conducted comprehensive analysis of the role of cuproptosis in ccRCC. We described the genomic and transcriptional alterations of 10 cuproptosis genes in ccRCC relative to normal tissues, and observed that methylation and SCNA might result in the aberrant expression of cuproptosis genes. Consistent with previous research [13, 52], most cuproptosis genes (FDX1, LIAS, LIPT1, DLD, DLAT, PDHA1, PDHB, MTF1, and GLS) correlated to more favorable OS outcome of ccRCC, with opposite effect of CDKN2A, indicating their critical implications in ccRCC prognosis.

Based on the transcriptome profiling of cuproptosis genes, we defined the three cuproptosis subtypes (C1 (moderate cuproptosis), C2 (low cuproptosis), and C3 (high cuproptosis)) in ccRCC. NTP approach demonstrated that the cuproptosis-based classification could be effectively repeated in the TCGA dataset and externally verified in the E-MTAB-1980 dataset, proving that this classification was highly repeatable. OS was best in C3, moderate in C1, and worst in C2, indicating that cuproptosis might contribute to favorable survival outcome of ccRCC. Large-scale genomics research has characterized global somatic alteration features in ccRCC, and their relationships with prognosis [53]. Chromosome 3p loss leads to inactivation of several tumor suppressor genes (VHL, PBRM1, etc.), which has been defined as an early driver event in ccRCC [54]. The heterogeneity in genomic alterations occurred in the three cuproptosis subtypes. C1 exhibited higher SCNA, CTA score, homologous recombination defects, and intratumor heterogeneity than others.

The tumor microenvironment is a complex ecosystem comprising heterogeneous cell types. The complex interaction between renal cancer cells and the surrounding tumor microenvironment results in the remarkable intratumoral heterogeneity of ccRCC. In addition, renal cell carcinoma is one of the most immune-infiltrated tumors [55, 56]. Emerging evidence suggests that the activation of specific metabolic pathway have a role in regulating angiogenesis and inflammatory signatures [57, 58]. Features of the tumor microenvironment heavily affect disease biology and may affect responses to systemic therapy [59]. Accumulating evidence proves the essential role of cuproptosis in tumor immunity [60–62], but the mechanisms of cuproptosis molecules in ccRCC remain indistinct. Our evidence indicated the high correlation of cuproptosis subtypes with the tumor microenvironment. For example, subtype C1 and C2 were corresponding to a high immune cell infiltration, while C3 accompanied by an immune-desert type.

Moreover, patients in C1 benefited more from anti-PD-1 immunotherapy. Additionally, C1 patients were most sensitive to pazopanib, and sorafenib, and C2 patients had the highest sensitivity to Sunitinib. Altogether, this cuproptosis-based classification might assist therapeutic options for ccRCC patients.

To improve prognostic outcome of ccRCC, understanding of the molecular underpinnings within the spectrum of ccRCC progression is required. We defined the Cuscore system to quantify cuproptosis of individual ccRCC patients. High Cuscore group presented the remarkable survival advantage. Additionally, the Cuscore was highly associated with immunogenomic features, and post-transcriptional events that contributed to ccRCC development. Several potential compounds and druggable targets (NMU, RARRES1) were determined for patients with low Cuscore. NMU is a neuropeptide implicated in energy homeostasis and tumor progression. VHL inactivation leads to remarkable upregulation of NMU in renal cancer cells, and NMU facilitates renal cancer progression through an autocrine effect [63]. RARRES1 can regulate podocyte function, and its expression is up-regulated in glomerular diseases and correlated to disease progression [64]. Highly expressed RARRES1 results in podocyte apoptosis by autocrine and paracrine effects [65]. Of note, RARRES1 expression is stronger in high grade versus low-grade RCC, and RARRES1-positive patients have poorer OS [66]. Combining the existing evidence, NMU and RARRES1 might be potential druggable targets of ccRCC patients with low Cuscore.

Of course, we recognize some limitations of this study. This is a retrospective study. Although we observed the heterogeneity in cuproptosis in as many ccRCC patients as possible, multicenter clinical cohorts are required for further analysis and validation. Our data suggested that cuproptosis may play a crucial role in ccRCC, but the molecular mechanisms involved are still understudied. This is a study of basic research and more in vitro and in vivo studies are necessary in the future.

## Conclusion

Altogether, our findings proposed three different cuproptosis subtypes that provided a novel insight into the relationships of cuproptosis with clinical, molecular, and immune characteristics of ccRCC. In addition, we generated the reliable Cuscore that enabled to accurately predict the survival outcomes and immunotherapeutic response in patients with ccRCC. This work provides a roadmap for patients’ stratification, and may help inform personalized follow-up and individualized decision-making strategies for ccRCC immunotherapy, and advance the development of precision immuno-oncology.

## Supplementary Information

**Additional file 1: Figure S1.** Unsupervised clustering analysis of ccRCC samples based on the transcriptome profiling of cuproptosis genes in the TCGA-KIRC cohort. (A) Consensus CDF plot displays the consensus distribution at different k. (B) Relative alterations in area under CDF curves. (C) Item tracking shows the consensus clusters (column) across different k (row).

**Additional file 2: Table S1.** The detailed clinicopathological information of TCGA and the E-MTAB-1980 cohorts.

**Additional file 3: Table S2.** The marker genes of 22 tumor-infiltrating immune cell types and two stromal components.

**Additional file 4: Table S3.** The list of 75 immunomodulators.

**Additional file 5: Table S4.** The information of three cuproptosis subtypes in TCGA cohort.

**Additional file 6: Table S5.** The top 200 of up-regulated biomarkers of each cuproptosis subtype.

**Additional file 7: Table S6.** The list of 95 cuproptosis-related genes.

**Additional file 8: Table S7.** Univariate Cox regression results of 95 cuproptosis-related genes.

**Additional file 9: Table S8.** Sixty-nine miRNAs with differential expression between low and high Cuscore groups.

## Data Availability

The datasets analyzed in this study are available in the TCGA (https://portal.gdc.cancer.gov/), ICGC (https://dcc.icgc.org/), and GEO (https://www.ncbi.nlm.nih.gov/geo/) database.

## Abbreviations

ccRCC: Clear cell renal cell carcinoma
RCC: Renal cell carcinoma
TCGA: The Cancer Genome Atlas
TPM: Transcripts per million
SCNA: Somatic copy-number alteration
miRNA: MicroRNA
GO: Gene Ontology
KEGG: Kyoto Encyclopedia of Genes and Genomes
GSEA: Gene set enrichment analysis
FDR: False discovery rate
GSVA: Gene set variation analysis
fGSEA: Fast GSEA
NTP: Nearest template prediction
SNV: Single-nucleotide variant
TMB: Tumor mutation burden
CTA: Cancer-testis antigen
mRNAsi: MRNA expression-based stemness index
CCLs: Cancer cell lines
GDSC: Genomics of drug sensitivity in cancer
CTRP: Cancer therapeutics response portal
CCLE: Cancer cell line encyclopedia
ssGSEA: Single-sample GSEA
SubMap: Subclass mapping
Cuscore: Cuproptosis score
DEGs: Differentially expressed genes
PCs: Principal components
K-M: Kaplan–Meier
OS: Overall survival
ROC: Receiver operating characteristic
AUC: Area under the curve
PCA: Principal component analysis
CDF: Cumulative distribution function

## References

[1] Motzer RJ, Jonasch E, Agarwal N (2022). Kidney cancer, Version 3.2022, NCCN clinical practice guidelines in oncology. J Natl Compr Canc Netw.

[2] Hu J, Chen Z, Bao L (2020). Single-cell transcriptome analysis reveals intratumoral heterogeneity in ccRCC, which results in different clinical outcomes. Mol Ther.

[3] Long Z, Sun C, Tang M (2022). Single-cell multiomics analysis reveals regulatory programs in clear cell renal cell carcinoma. Cell Discov.

[4] Udayakumar D, Zhang Z, Xi Y (2021). Deciphering intratumoral molecular heterogeneity in clear cell renal cell carcinoma with a radiogenomics platform. Clin Cancer Res.

[5] Di Lascio G, Sciarra A, Giudice F (2022). Which factors can influence post-operative renal function preservation after nephron-sparing surgery for kidney cancer: a critical review. Cent Eur J Urol.

[6] Ferro M, Musi G, Marchioni M (2023). Radiogenomics in renal cancer management-current evidence and future prospects. Int J Mol Sci.

[7] Ferro M, Crocetto F, Barone B (2023). Artificial intelligence and radiomics in evaluation of kidney lesions: a comprehensive literature review. Ther Adv Urol.

[8] Gui CP, Wei JH, Chen YH (2021). A new thinking: extended application of genomic selection to screen multiomics data for development of novel hypoxia-immune biomarkers and target therapy of clear cell renal cell carcinoma. Brief Bioinform.

[9] Linehan WM, Ricketts CJ (2019). The Cancer Genome Atlas of renal cell carcinoma: findings and clinical implications. Nat Rev Urol.

[10] Ge EJ, Bush AI, Casini A (2022). Connecting copper and cancer: from transition metal signalling to metalloplasia. Nat Rev Cancer.

[11] Gupte A, Mumper RJ (2009). Elevated copper and oxidative stress in cancer cells as a target for cancer treatment. Cancer Treat Rev.

[12] Zhang H, Meltzer P, Davis S (2013). RCircos: an R package for Circos 2D track plots. BMC Bioinform.

[13] Bian Z, Fan R, Xie L (2022). A novel cuproptosis-related prognostic gene signature and validation of differential expression in clear cell renal cell carcinoma. Genes (Basel).

[14] Xu S, Liu D, Chang T (2022). Cuproptosis-associated lncRNA establishes new prognostic profile and predicts immunotherapy response in clear cell renal cell carcinoma. Front Genet.

[15] Tsvetkov P, Coy S, Petrova B (2022). Copper induces cell death by targeting lipoylated TCA cycle proteins. Science.

[16] Mayakonda A, Lin DC, Assenov Y (2018). Maftools: efficient and comprehensive analysis of somatic variants in cancer. Genome Res.

[17] Mermel CH, Schumacher SE, Hill B (2011). GISTIC2.0 facilitates sensitive and confident localization of the targets of focal somatic copy-number alteration in human cancers. Genome Biol.

[18] Gu Z, Hübschmann D (2021). Make interactive complex heatmaps in R. Bioinformatics.

[19] Subramanian A, Tamayo P, Mootha VK (2005). Gene set enrichment analysis: a knowledge-based approach for interpreting genome-wide expression profiles. Proc Natl Acad Sci U S A.

[20] Wu T, Hu E, Xu S (2021). clusterProfiler 4.0: a universal enrichment tool for interpreting omics data. Innovation (Camb)..

[21] Hänzelmann S, Castelo R, Guinney J (2013). GSVA: gene set variation analysis for microarray and RNA-seq data. BMC Bioinformatics.

[22] Liberzon A, Birger C, Thorvaldsdóttir H (2015). The Molecular Signatures Database (MSigDB) hallmark gene set collection. Cell Syst.

[23] Wilkerson MD, Hayes DN (2010). ConsensusClusterPlus: a class discovery tool with confidence assessments and item tracking. Bioinformatics.

[24] Ritchie ME, Phipson B, Wu D (2015). limma powers differential expression analyses for RNA-sequencing and microarray studies. Nucleic Acids Res.

[25] Hoshida Y (2010). Nearest template prediction: a single-sample-based flexible class prediction with confidence assessment. PLoS ONE.

[26] Thorsson V, Gibbs DL, Brown SD (2018). The immune landscape of cancer. Immunity.

[27] Malta TM, Sokolov A, Gentles AJ (2018). Machine learning identifies stemness features associated with oncogenic dedifferentiation. Cell.

[28] Yang W, Soares J, Greninger P (2013). Genomics of Drug Sensitivity in Cancer (GDSC): a resource for therapeutic biomarker discovery in cancer cells. Nucleic Acids Res.

[29] Yu C, Mannan AM, Yvone GM (2016). High-throughput identification of genotype-specific cancer vulnerabilities in mixtures of barcoded tumor cell lines. Nat Biotechnol.

[30] Barretina J, Caponigro G, Stransky N (2012). The cancer cell line encyclopedia enables predictive modelling of anticancer drug sensitivity. Nature.

[31] Maeser D, Gruener RF, Huang RS (2021). oncoPredict: an R package for predicting in vivo or cancer patient drug response and biomarkers from cell line screening data. Brief Bioinform.

[32] Geeleher P, Cox N, Huang RS (2014). pRRophetic: an R package for prediction of clinical chemotherapeutic response from tumor gene expression levels. PLoS ONE.

[33] Charoentong P, Finotello F, Angelova M (2017). Pan-cancer immunogenomic analyses reveal genotype-immunophenotype relationships and predictors of response to checkpoint blockade. Cell Rep.

[34] Yoshihara K, Shahmoradgoli M, Martínez E (2013). Inferring tumour purity and stromal and immune cell admixture from expression data. Nat Commun.

[35] Liu Z, Zhang Y, Shi C (2021). A novel immune classification reveals distinct immune escape mechanism and genomic alterations: implications for immunotherapy in hepatocellular carcinoma. J Transl Med.

[36] Hoshida Y, Brunet JP, Tamayo P (2007). Subclass mapping: identifying common subtypes in independent disease data sets. PLoS ONE.

[37] Chevrier S, Levine JH, Zanotelli VRT (2017). An immune atlas of clear cell renal cell carcinoma. Cell.

[38] Wang Y, Zhang L, Zhou F (2022). Cuproptosis: a new form of programmed cell death. Cell Mol Immunol.

[39] Meo N, Lasorsa F, Rutigliano M (2023). The dark side of lipid metabolism in prostate and renal carcinoma: novel insights into molecular diagnostic and biomarker discovery. Expert Rev Mol Diagn.

[40] Lucarelli G, Loizzo D, Franzin R (2019). Metabolomic insights into pathophysiological mechanisms and biomarker discovery in clear cell - renal cell carcinoma. Expert Rev Mol Diagn.

[41] Meo N, Lasorsa F, Rutigliano M (2022). Renal cell carcinoma as a metabolic disease: an update on main pathways, potential biomarkers, and therapeutic targets. Int J Mol Sci.

[42] Marco S, Torsello B, Minutiello E (2022). The cross-talk between Abl2 tyrosine kinase and TGFβ1 signalling modulates the invasion of clear cell Renal Cell Carcinoma cells. FEBS Lett.

[43] Bianchi C, Meregalli C, Bombelli S (2017). The glucose and lipid metabolism reprogramming is grade dependent in clear cell renal cell carcinoma primary cultures and is targetable to modulate cell viability and proliferation. Oncotarget.

[44] Ragone R, Sallustio F, Piccinonna S (2016). Renal cell carcinoma: a study through NMR-based metabolomics combined with transcriptomics. Diseases.

[45] Lucarelli G, Galleggiante V, Rutigliano M (2015). Metabolomic profile of glycolysis and the pentose phosphate pathway identifies the central role of glucose-6-phosphate dehydrogenase in clear cell-renal cell carcinoma. Oncotarget.

[46] Lucarelli G, Rutigliano M, Sallustio F (2018). Integrated multi-omics characterization reveals a distinctive metabolic signature and the role of NDUFA4L2 in promoting angiogenesis, chemoresistance, and mitochondrial dysfunction in clear cell renal cell carcinoma. Aging.

[47] Bombelli S, Torsello B, Marco S (2020). 36-kDa Annexin A3 isoform negatively modulates lipid storage in clear cell renal cell carcinoma cells. Am J Pathol.

[48] Lucarelli G, Rutigliano M, Loizzo D (2022). MUC1 tissue expression and its soluble form CA 15–3 identify a clear cell renal cell carcinoma with distinct metabolic profile and poor clinical outcome. Int J Mol Sci.

[49] Xie J, Yang Y, Gao Y, He J (2023). Cuproptosis: mechanisms and links with cancers. Mol Cancer.

[50] Xiong C, Ling H, Hao Q, Zhou X (2023). Cuproptosis: p53-regulated metabolic cell death?. Cell Death Differ.

[51] Ke D, Zhang Z, Liu J (2023). Ferroptosis, necroptosis and cuproptosis: Novel forms of regulated cell death in diabetic cardiomyopathy. Front Cardiovasc Med.

[52] Khouja HI, Ashankyty IM, Bajrai LH (2022). Multi-staged gene expression profiling reveals potential genes and the critical pathways in kidney cancer. Sci Rep.

[53] Patel SA, Hirosue S, Rodrigues P (2022). The renal lineage factor PAX8 controls oncogenic signalling in kidney cancer. Nature.

[54] Qu Y, Feng J, Wu X (2022). A proteogenomic analysis of clear cell renal cell carcinoma in a Chinese population. Nat Commun.

[55] Vuong L, Kotecha R, Voss M, Hakimi A (2019). Tumor microenvironment dynamics in clear-cell renal cell carcinoma. Cancer Discov.

[56] Tamma R, Rutigliano M, Lucarelli G (2019). Microvascular density, macrophages, and mast cells in human clear cell renal carcinoma with and without bevacizumab treatment. Urol Oncol.

[57] Stefano N, Lucarelli G, Spadaccino F (2020). PTX3 modulates the immunoflogosis in tumor microenvironment and is a prognostic factor for patients with clear cell renal cell carcinoma. Aging.

[58] Lucarelli G, Rutigliano M, Ferro M (2017). Activation of the kynurenine pathway predicts poor outcome in patients with clear cell renal cell carcinoma. Urol Oncol.

[59] Ghini V, Laera L, Fantechi B (2020). Metabolomics to assess response to immune checkpoint inhibitors in patients with non-small-cell lung cancer. Cancers.

[60] Lv H, Liu X, Zeng X (2022). Comprehensive analysis of cuproptosis-related genes in immune infiltration and prognosis in melanoma. Front Pharmacol.

[61] Song Q, Zhou R, Shu F, Fu W (2022). Cuproptosis scoring system to predict the clinical outcome and immune response in bladder cancer. Front Immunol.

[62] Zhang Z, Zeng X, Wu Y (2022). Cuproptosis-related risk score predicts prognosis and characterizes the tumor microenvironment in hepatocellular carcinoma. Front Immunol.

[63] Harten SK, Esteban MA, Shukla D (2011). Inactivation of the von Hippel-Lindau tumour suppressor gene induces Neuromedin U expression in renal cancer cells. Mol Cancer.

[64] Chen A, Feng Y, Lai H (2020). Soluble RARRES1 induces podocyte apoptosis to promote glomerular disease progression. J Clin Invest.

[65] Chen A, Lee K, He JC (2021). Autocrine and paracrine effects of a novel podocyte gene, RARRES1. Kidney Int.

[66] Zimpfer A, Dammert F, Glass A (2016). Expression and clinicopathological correlations of retinoid acid receptor responder protein 1 in renal cell carcinomas. Biomark Med.

