# Supplementary figures and images for: Multi-omics analysis uncovers clinical, immunological, and pharmacogenomic implications of cuproptosis in clear cell renal cell carcinoma
Source: Eur J Med Res. 2023 Jul 22;28:248. doi: 10.1186/s40001-023-01221-4 (PMC10362584; doi:10.1186/s40001-023-01221-4)

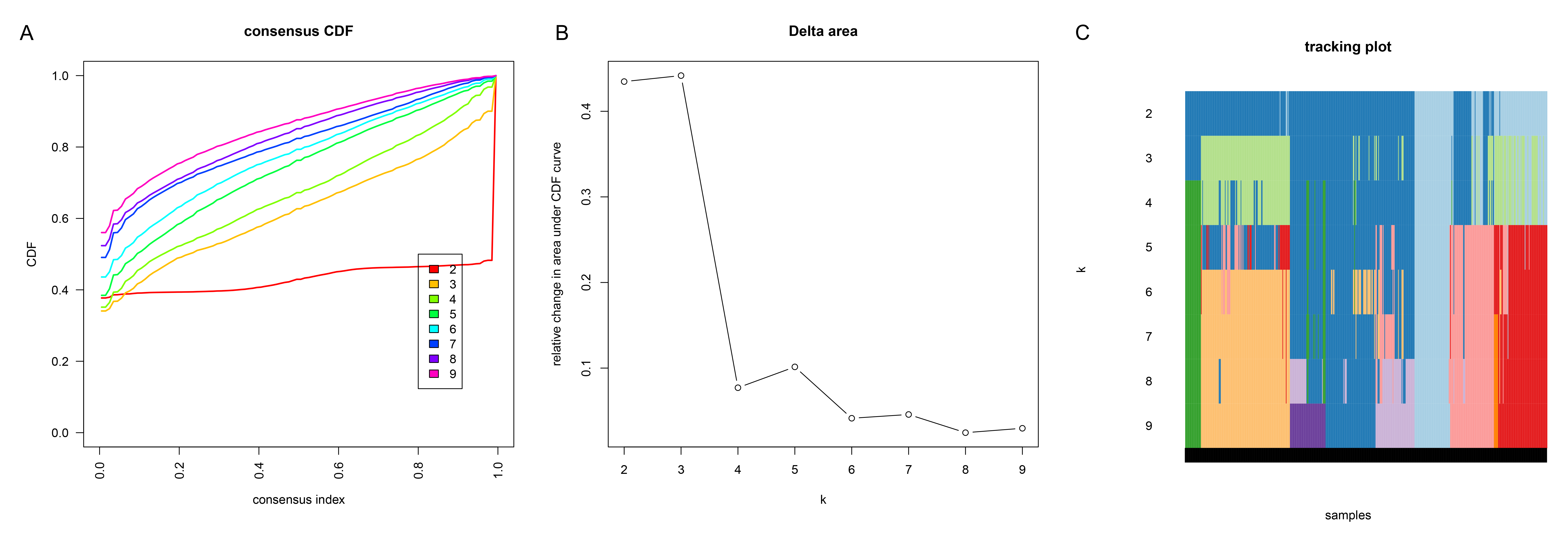

Supplement: Supplementary file 1 — Additional file 1: Figure S1. Unsupervised clustering analysis of ccRCC samples based on the transcriptome profiling of cuproptosis genes in the TCGA-KIRC cohort. (A) Consensus CDF plot displays the consensus distribution at different k. (B) Relative alterations in area under CDF curves. (C) Item tracking shows the consensus clusters (column) across different k (row). [file 40001_2023_1221_MOESM1_ESM.tif]
